# Lipid-based nanocarriers for enhanced gentamicin delivery: a comparative study of liquid crystal nanoparticles and liposomes against *Escherichia coli* biofilms

**DOI:** 10.1007/s13346-025-01890-0

**Published:** 2025-06-12

**Authors:** Anam Ahsan, Timothy J. Barnes, Nicky Thomas, Santhni Subramaniam, Clive A. Prestidge

**Affiliations:** Centre for Pharmaceutical Innovation, UniSA Clinical and Health Sciences, University of South Australia, Adelaide, South Australia 5000 Australia

**Keywords:** Lipid nanoparticles, Liquid crystal nanoparticles, Liposomes, *E. coli*, Biofilm, Gentamicin, In vitro

## Abstract

Conventional antibiotic therapies often fail to eradicate bacterial biofilms due to limited penetration, altered microenvironments, and the presence of persister cells, contributing to persistent and recurrent infections. As a result,the growing threat of antibiotic-resistant bacteria, particularly those forming biofilms, underscores the urgent need for alternative therapeutic strategies. Lipid-based drug delivery systems have emerged as effective nanocarriers for antimicrobials, offering a promising strategy to combat bacterial biofilms due to their biomimetic properties, biocompatibility, and ability to navigate the complex physical, chemical, and biological barriers posed by biofilms. In this study, we compared liquid crystal nanoparticles (LCNPs) and liposomes as delivery systems for gentamicin (GEN) against *Escherichia coli (E. coli*) (ATCC 25922 and ATCC 35218) in both planktonic and biofilm forms. Transmission electron microscopy analysis confirmed the particle size of GEN-loaded LCNPs (~ 200 nm) and GEN-loaded liposomes (~ 160 nm), with cubic-shaped LCNPs and lipid bilayer-structured liposomes which remained stable over three weeks at 4ºC. Loading GEN into lipid-based nanoparticles resulted in a two-fold reduction in minimum inhibitory concentration values, without significantly altering the minimum bactericidal concentration. Notably, GEN-LCNPs led to a significant fourfold (for *E. coli* ATCC 25922) and threefold (for *E. coli* ATCC 35218) reduction in inhibitory concentrations in biofilm states compared to unformulated GEN, achieving a minimum biofilm inhibitory concentration (MBIC) of 50 μg/mL and 100 μg/mL (P < 0.0001), respectively. In contrast, liposomes showed only a twofold reduction in MBIC values (100–150 μg/mL) for both bacterial biofilms. GEN-loaded LCNPs also reduced the *E. coli* ATCC 25922 colony-forming unit count by 5000-fold and 4000-fold, while liposomes with similar particle size did not significantly improve GEN’s antimicrobial activity. Moreover, LCNPs improved GEN efficacy regardless of particle size or Pluronic^®^ concentration. In conclusion, our findings suggest that GEN-loaded LCNPs demonstrated superior antimicrobial efficacy against *E. coli* biofilms compared to liposomes, highlighting their potential as effective nanocarriers for combating antibiotic-resistant infections.

**Graphical Abstract:**

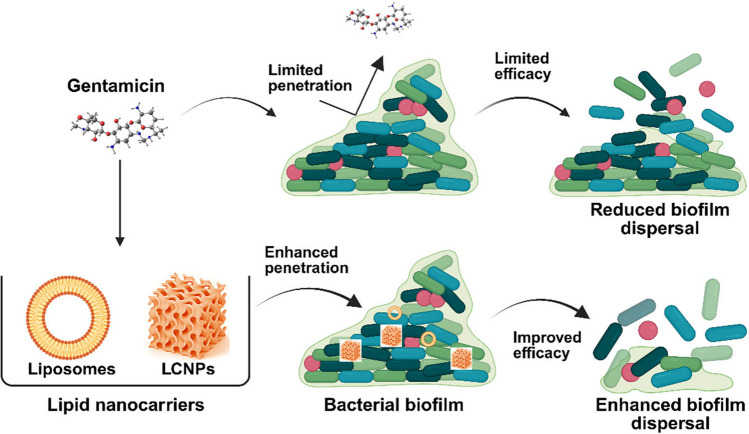

## Introduction

*Escherichia coli (E. coli)* is a commensal and opportunistic pathogen in mammals and birds [1–3]. Most *E. coli* strains are harmless and reside in the human gut as part of the normal microbiota by contributing to intestinal health, aiding in digestion, producing essential vitamins like vitamin K, and preventing colonisation by harmful pathogens through competitive exclusion [4]. In humans, its prevalence exceeds 90%, with concentrations of 10^7^ to 10^9^ colony-forming units per gram of faeces [5]. The scientific literature provides an abundance information on the nature and behaviour of *E. coli* [6–9]. *E. coli* strains can cause extraintestinal infections, including urinary tract infections (UTIs), various intra-abdominal, respiratory, skin and soft tissue infections, bacteraemia, and neonatal meningitis (NBM). They can also lead to intestinal infections, such as various forms of diarrhea, including hemolytic uremic syndrome (HUS).These infections can be ubiquitous (UTIs) [10], with high morbidity (renal failure in children with HUS [11], neurological sequelae in NBM [12]) and high mortality (about 15% for bacteraemia) [13].

Moreover, *E. coli* serves as a major reservoir of antimicrobial resistance genes, contributing to therapeutic failures in both human and veterinary medicine. It currently ranks third among the 12 antibiotic-resistant “priority pathogens” identified by the WHO, underscoring its critical impact on public health. [14, 15]. Recently, *E. coli* has been reported as a significant environmental threat, as it facilitates the spread of antibiotic resistance genes (ARGs) through integrons, transmissible plasmids, and transposons, endangering environmental safety and human health [16]. In the last decade, a growing number of resistance genes have been detected in *E. coli* isolates, with the majority being acquired through horizontal gene transfer [17]. These resistance mechanisms enhance *E. coli’s* ability to form biofilms, further protecting bacterial communities from antibiotics and immune responses, making infections even more difficult to eradicate.

*E. coli* biofilms are composed of bacterial colonies embedded in an extracellular polymeric substance (EPS) matrix, which shields microorganisms from harsh environmental conditions and contributes to infection. Certain pathogenic strains of *E. coli* are significant contributors to morbidity and mortality causing biofilm associated infections in chronic wounds, intestines, and the heart (e.g., endocarditis) [18].They are also commonly associated with medical devices e.g., urinary and intravascular catheters, prosthetic grafts, prosthetic shunts and joints [19]. Clinically, biofilm-associated bacterial infections tend to be chronic and challenging to treat as the matrix-embedded bacteria exhibit increased tolerance to antibiotics and host immune responses [20]. The extracellular polymeric substance (EPS) surrounding biofilm bacteria provides a protective barrier, leading to up to a 10,000-fold increase in antibiotic tolerance compared to planktonic bacteria [21]. This is due to the EPS matrix acting as a diffusion barrier, delaying the penetration of antimicrobial agents into the biofilm. The reduced growth and reproduction rate of bacteria within the biofilm further limit the drug uptake [22]. As a result, drug concentrations within biofilms remain below therapeutic levels, which can lead to significantly reduced efficacy while promoting antimicrobial resistance [23]. Additionally, the robust mechanical integrity of the biofilm matrix makes their removal from the human body challenging, often necessitating aggressive treatments that can cause excessive side effects or even require surgical intervention. [24]. Moreover, the integrated behaviour of bacteria within biofilms facilitates interactions and communication through quorum sensing, which enhances reproduction, survival, and further resistance to treatment [21]. While individual bacteria in deeper biofilm layers show reduced growth due to limited nutrients and oxygen, the biofilm as a whole benefits from coordinated behaviors like quorum sensing. These collective strategies enhance survival, virulence, and antibiotic resistance at the community level [25].Furthermore, the limited availability of effective antibiotics for biofilm treatment, combined with the growing threat of bacterial antimicrobial resistance, presents a significant clinical challenge [26].

**Fig. 1:**
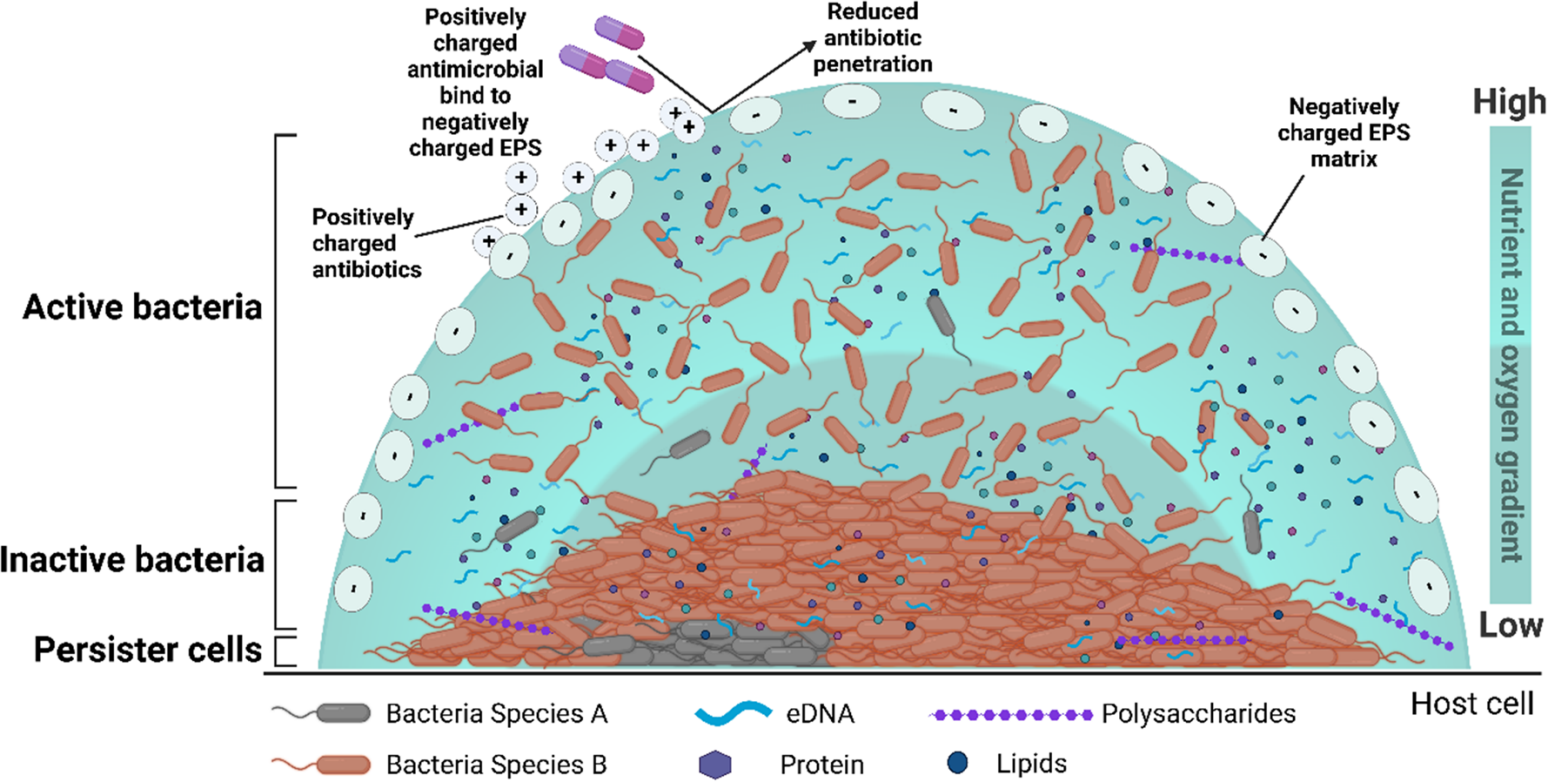
An illustration of a bacterial biofilm depicting the limited penetration of positively charged antibiotics, such as gentamicin, into the negatively charged EPS matrix. The figure was created using BioRender.com

**Fig. 2:**
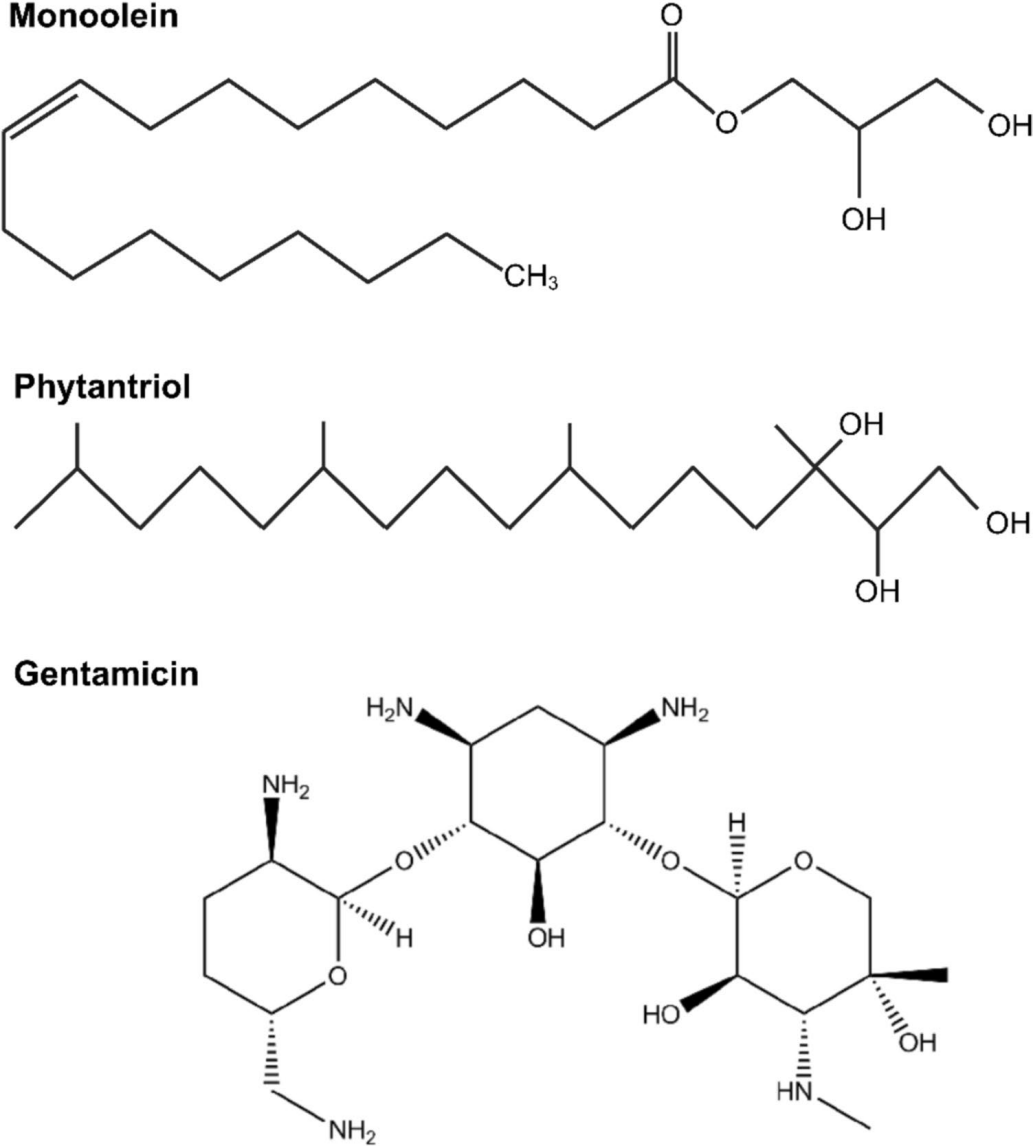
Chemical structures of monoolein, phytantriol and gentamicin. These structures were drawn with Biorender.com

Gentamicin (GEN) is a broad-spectrum aminoglycoside antibiotic that effectively inhibits the growth of various Gram-positive and Gram-negative pathogens [27, 28]. It has been administered systemically to treat infections such as those of the biliary tract, urinary tract, soft tissues, meningitis, and endocarditis. Additionally, it is used topically to manage localised infections, including seborrheic dermatitis, impetigo, and superficial ocular infections [29]. Interestingly, GEN is one of the most widely used antibiotics in controlled-release devices and has good solubility and high temperature stability. *E. coli* is one of the clinical pathogens included in the antimicrobial spectrum of aminoglycoside antibiotics [30]. However, GEN has limited efficacy against *E. coli* biofilms because the antibiotic is unable to penetrate the biofilm [31]. As a cationic molecule, GEN binds to negatively charged polysaccharides in the biofilm matrix, preventing it from penetrating the inner bacterial community [32] (Figs. 1 and 2). GEN exerts its antimicrobial effects by inhibiting protein synthesis and damaging the bacterial cell membrane [33]. As a concentration-dependent antibiotic, achieving sufficiently high concentrations is crucial for its effectiveness [33]. However, this requirement poses significant toxicity risks, including neurotoxicity, nephrotoxicity, ototoxicity, and neuromuscular blockade, particularly when administered parenterally at high doses for extended periods. Additionally, prolonged use contributes to the development of drug-resistant bacterial strains [34].

Recently, there has been increasing interest in reformulating antibiotics to enhance their effectiveness against biofilm-associated infections [31]. Various nanocarriers, including polymeric nanoparticles, mesoporous silica nanoparticles, hydrogels, dendrimers, and lipid nanoparticles, have been explored for delivering antimicrobial agents [23]. Among these, lipid-based drug delivery systems (LBDDS), such as liposomes and liquid crystal nanoparticles (LCNPs), are widely regarded as effective carriers due to their biomimetic properties, biocompatibility, and ability to overcome the physical, chemical, and biological barriers posed by bacteria [23]. LBDDS have also been demonstrated to improve the efficacy of existing antibiotics such as vancomycin, tobramycin, meropenem, levofloxacin, ceftazidime, and azithromycin [35–38].

Liposomes consist of a phospholipid bilayer enclosing an aqueous core, enabling the encapsulation of amphiphilic and hydrophilic drugs, while the lipid bilayer itself accommodates lipophilic drugs [23]. Beyond liposomes, lipids can self-assemble into more structured, non-lamellar 3D mesophases, such as bicontinuous cubic (cubosomes) and inverse hexagonal phases (hexasomes). These structures, collectively known as lipid-based cubic and hexagonal nanoparticles (LCNPs), exhibit increased bilayer curvature, enhancing their potential in drug delivery [39]. Often referred to as liquid crystals, LCNPs provide a larger surface area within the lipid bilayer, allowing for the encapsulation of a wider range of amphiphilic, hydrophilic, and lipophilic drugs [23]. Their lipid core effectively traps hydrophobic drugs, while the outer shell improves solubility and stability Monoolein (MO) and phytantriol (PHY) (Fig. 2) are the most commonly used lipids for forming liquid crystals, both of which are generally recognised as safe (GRAS) and widely utilised in cosmetics and food products [40, 41]. Although, MO is often preferred over PHY due to its lower propensity to induce haemolysis at clinically relevant concentrations [42], the inclusion of PHY in this study is likely driven by formulation and mechanistic considerations rather than direct clinical translation. As a monoglyceride, MO contains a lipase-labile ester functional group, which has historically posed challenges for oral drug delivery using MO-LCNPs. Gastrointestinal digestion of MO can lead to precipitation, reducing the absorption of poorly water-soluble drugs [43]. Conversely, PHY is known to form highly stable and well-defined cubic phase nanostructures, often resulting in more robust LCNPs with desirable properties such as enhanced drug encapsulation, controlled release, and structural stability [41].

Liposomal formulations represent one of the most effective strategies for antibiotic delivery, as they can fuse with bacterial cell membranes, improve targeting, and enhance antibiotic concentrations within bacterial cells. [44]. Numerous studies have reported the successful encapsulation of antibiotics in liposomes using various lipid compositions [45]. For instance, an inhaled liposomal formulation, has been approved for delivering amikacin to treat *Mycobacterium avium* complex (MAC) lung disease. However, its use for targeting *P. aeruginosa* biofilms in cystic fibrosis is still under clinical investigation. [46]. Beyond their fusogenic properties, the submicron size and surface characteristics of liposomes improve targeted antibiotic delivery to infection sites, enhancing the penetration of aminoglycosides into biofilms and intracellular bacterial infections [39]. While on the contrary, LCNPs are still largely in the research and development phase for many therapeutic applications. They have been explored for the targeted delivery of antibiotics, peptides, antibodies, siRNA, DNA, and vaccines [47, 48]. By enhancing drug solubility and stability, LCNPs can improve the bioavailability of poorly water-soluble drugs. Additionally, they enable controlled and sustained drug release, reducing dosing frequency and enhancing therapeutic outcomes [47]. Our group previously developed MO-LCNPs as a drug delivery system for antimicrobial drugs, where drug release was triggered by the presence of bacteria. [41]. Bacterial lipase digestion of MO-LCNPs led to an 82-fold increase in the release rate of rifampicin and a sevenfold increase for alginate lyase antimicrobials. In contrast, small hydrophilic antibiotics like ciprofloxacin hydrochloride and non-digestible PHY-LCNPs did not exhibit this response [41]. LCNPs have been shown to form a patchy coating on biofilms, which enhances the activity of the cationic antibiotic tobramycin against *P. aeruginosa*, without impacting the activity of ciprofloxacin, which can freely penetrate biofilms [49].

Since antibiotics that already penetrate biofilms easily showed no added benefit when encapsulated in LCNPs, producing effects similar to those of the unformulated (neat) antibiotic [49], the current study shifts focus to the cationic antibiotic GEN, which cannot penetrate biofilms, though planktonic *E. coli* is susceptible to it. In this study, we compare MO- and PHY-LCNPs for GEN delivery against *E. coli* biofilms, aiming to improve biofilm penetration and enhance GEN efficacy. To further assess the effects of GEN-LCNPs, we compared them to GEN-liposomal formulations in several in vitro* E. coli* biofilm models to evaluate their relative effectiveness in biofilm treatment. Additionally, the Pluronic^®^ concentration in GEN-loaded MO-LCNPs was varied to study the influence of stabilizer coating on antimicrobial activity.

## Materials & methods

### Materials

Monoolein, supplied as Myverol (p/n: 5D01253), was generously donated by Kerry Ingredients and Flavours (Egham, Surrey, UK), while phytantriol was provided by DSM (Heerlen, The Netherlands). Gentamicin and USP-grade methanol were sourced from ChemSupply (Port Adelaide, South Australia, Australia). DPPG and DPPC lipids were obtained from Merck Life Sciences (Bayswater, VIC, Australia). Tryptic soy agar (TSA), Brain Heart Infusion (BHI) broth, Luria–Bertani (LB) broth, and Alamar Blue dye were purchased from Oxoid Limited (Thermo Fisher Scientific Australia Pty Ltd., Scoresby, Victoria, Australia). Other chemicals, including Pluronic^®^ F-127, propylene glycol, acetonitrile (HPLC grade), chloroform (> 99.9% HPLC), formic acid (HPLC grade), 0.01 M phosphate-buffered saline (PBS) tablets, methanol, crystal violet dye, and acetic acid were procured from Sigma-Aldrich (St. Louis, MO, USA). Milli-Q grade water, purified using a Direct Water Purification System (Millipore, MA, USA), was used throughout the study.

### Fabrication of liquid crystal nanoparticles (LCNPs)

LCNPs were prepared using the hydrotrope dilution method [50]. In a glass scintillation vial, MO (100 mg) or PHY (100 mg) was mixed with propylene glycol (270 µL) and Pluronic^®^ F-127 (15 mg) before being dissolved in 10 mL of chloroform. The chloroform was then evaporated under a nitrogen stream to form a lipid film. This lipid film was hydrated by adding 100 µL of 0.9% w/v NaCl containing 250 mg/mL GEN, followed by vortexing for 30 s. Finally, the lipid mixture was diluted to a total volume of 5 mL with Milli-Q water and vortexed for approximately 2 min until a uniform milky emulsion was obtained.

### Fabrication of liposomes

The Nanoassemblr^®^ microfluidic device (Precision Nanosystems, Vancouver, BC, Canada) was used to prepare liposomes from a 1:1 mixture of 1,2-dipalmitoyl phosphatidylglycerol (DPPG) and 1,2-dipalmitoyl-sn-glycero-3-phosphocholine (DPPC) dissolved in methanol. Within the microfluidic system, 1.5 mL of an aqueous GEN solution was mixed with 1 mL of the DPPG: DPPC methanol solution at a total flow rate of 12 mL/min. The resulting liposomes were collected and stirred overnight at room temperature (25 ºC) to ensure the complete evaporation of any residual methanol [50].

### Physico-chemical characterisation

LCNP and liposome samples, diluted 1:1000 in water, were analysed at 25 °C using dynamic light scattering and phase analysis light scattering (Zetasizer Nano ZS, Malvern Panalytical, Worcestershire, UK) to determine particle size and zeta potential (ζ), respectively. Measurements were performed with a refractive index of 1.48. The built-in software calculated the mean hydrodynamic diameter (z-average), and particle size distribution (polydispersity index, PDI) based on 15 triplicate measurements, assuming a unimodal distribution.

### Nanoparticle tracking analysis

The particle concentration of LCNPs and liposomes, diluted 1:1000 in Milli-Q water, was determined using nanoparticle tracking analysis (NTA) with the NanoSight NS300 (Malvern Panalytical, Worcestershire, UK). The built-in software analysed four recorded videos of the samples at 25 °C, detecting an average of 31 particles per frame, with a syringe pump operating at a flow rate of 60 μL/s.

### Cryogenic transmission electron microscopy

The morphology of GEN-LCNPs and liposomes was examined using a Glacios 200 kV Cryo-TEM (Thermo Fisher Scientific™). In brief, 5 μL of the GEN-LCNP and liposome sample was applied to a 300-mesh copper grid and glow-discharged for 30 s. The sample was then vitrified in a liquid ethane/propane mixture and maintained at −180 °C during imaging. Micrographs were captured at 120 kV using a NANOSPRT15 camera (Thermo Fisher Scientific) with the microscope operating in bright-field mode.

### Fast Fourier Transform (FFT) analysis

FFT analysis of LCNPs was conducted using the built-in FFT function in Velox Software (Thermo Fisher Scientific). The images were exported as TIFF files. FFT (Fast Fourier Transform) analysis of LCNPs and liposomes is typically done to analyse their structural properties, such as their particle size distribution, shape, and internal organisation. By applying FFT, detailed information on the periodicity and symmetry of the nanoparticles can be obtained, which helps in understanding their behaviour in drug delivery systems [51].

### Stability of LCNPs and liposomes

GEN-LCNPs and GEN-liposomes (original formulation suspensions) were stored in glass scintillation vials at 4 °C. Their average particle size, PDI, and zeta potential were measured weekly over four weeks (days 7, 14, 21, and 28) and compared to freshly prepared formulations.

### Measurement of Pluronic^®^ F-127 coating thickness

MO-LCNPs were formulated with varying concentrations of Pluronic^®^ F-127 (0–6% w/v) to assess its impact on antimicrobial activity while maintaining a constant GEN loading. The original formulation contained 0.3% w/v Pluronic^®^ F-127 in the final composition. The variation in zeta potential caused by the adsorbed Pluronic^®^ layer on the MO-LCNP surface was used to estimate the F-127 coating thickness [52] based on the following equation:

1 $$tanh\left(\frac{ze\zeta }{4KT}\right)=tanh\left(\frac{ze{\Psi }_{0}}{4{KT}^{e}}\right)-\kappa \delta$$

where *z* represents the valence of the potential-determining ions, *e* is the electron charge, ζ is the zeta potential, *Ψ₀* is the surface potential, *δ* is the thickness of the adsorbed layer, and *1/κ* is the Debye length.

### Encapsulation efficiency and in vitro release studies

The total GENcontent in the formulations was determined indirectly using a pressure ultrafiltration stirred cell (Amicon, Merck Millipore, Bayswater, VIC, Australia) [49]. A 2.5 mL sample of the undiluted formulation was placed in the cell, which contained a pre-soaked ultrafiltration membrane made of Biomax polyethersulfone with a 500 kDa NMWL (Merck Millipore, Bayswater, VIC, Australia). The formulations were ultrafiltered under 100 kPa nitrogen gas pressure, resulting in a filtrate free of LCNPs and liposomes due to the particle size exclusion of the ultrafiltration membrane. The filtrate was quantified to verify the unloaded antibiotic portion, while thetotal GEN content was then quantified by solubilising the LCNPs and liposomes with 5% v/v Triton X (Sigma-Aldrich, St. Louis, MO, USA) Encapsulation efficiency was calculated indirectly by quantifying the free drug in the filtrate and subtracting it from the total GEN content, which was determined directly after solubilising the nanoparticles with Triton X. To improve accuracy, we incorporated Triton X-mediated solubilisation of lipid carriers to determine total GEN content and used validated ultrafiltration conditions to ensure effective separation of free drug. This approach has been widely used in nanoparticle studies and offers a reliable estimate of encapsulation efficiency when direct quantification is analytically limited [53].

GEN was quantified using LC–MS (Shimadzu, Kyoto, Japan) based on a modified version of the method previously described by Lucha et al*.* [54]. Separation was carried out on a Kinetex 1.7 μm C18 100 Å (50 × 30 mm) column (Torrance, USA), equipped with a column guard. The system was maintained at 40 °C, with an injection volume of 2 μL and a mobile phase flow rate of 0.5 mL/min. The mobile phase consisted of 0.07% (v/v) trifluoracetic acid (TFA) in water and 0.1% (v/v) formic acid in acetonitrile. Each sample was analysed within 4 min, with detection at a wavelength of 365 nm and a typical retention time of 0.32 min. Quantification was performed using a calibration curve with standards of known GEN concentrations (0.5–100 μg/mL, R^2^ = 0.9973, limit of quantification 0.1 μg/mL).

After determining the amount of GEN not loaded in LCNPs and liposomes (as described above), the preparations were diluted 1:5 with buffered media (0.01 M Phosphate Buffered Saline (PBS) buffer, pH 7.4) in a pressure ultrafiltration cell to assess GEN release at room temperature (25 ºC). The mixture was magnetically stirred, and 0.5 mL samples were collected at specific time points (0, 5, 10, 15, 30, 45, 60, 90, 120, and 240 min) by applying 100 kPa nitrogen gas. After each sample was withdrawn, the volume was immediately replenished with 0.5 mL of fresh PBS buffer. The filtered samples, free of LCNPs and liposomes, were analysed for GEN content to determine the release profile. Additionally, unformulated GEN at equivalent concentrations was also analysed for its release using the ultrafiltration cell.

### Antimicrobial evaluation against planktonic E. coli-minimum inhibitory concentration & minimum bactericidal concentration

The antimicrobial activity of GEN, either as an unformulated solution or loaded into MO-LCNPs, PHY-LCNPs, and liposomes, was evaluated in 96-well plates against two planktonic *E. coli* strains (ATCC 25922—a strong biofilm producer, and ATCC 35218—a moderate biofilm producer). A standard broth microdilution assay was conducted to compare the antimicrobial efficacy of the GEN formulations against these two biofilm-producing strains. Logarithmic phase growth suspensions of both *E. coli* strains were prepared by diluting overnight cultures to an OD_600_ of 0.10 ± 0.02 (1 × 10^8^ CFU/mL) in Muller Hinton broth and adding them to the wells of a 96-well plate. Bacteria were mixed 1:1 with serial dilutions of GEN, ranging from 0.03–16 µg/mL. Equivalent concentrations of LCNPs and liposomes with GEN loading were also tested. After incubation at 37 °C for 18 h, the inhibitory concentration was determined by measuring the OD_600_ value of the last clear well, which was comparable to the sterile media control. The assay was performed in quadruplicate.

To determine the minimum bactericidal concentration (MBC), following the minimum inhibitory concentration (MIC) determination after 18 h of incubation, 0.01 mL was taken from the MIC well, from the turbid well on the right, and from the next two clear wells on the left. These samples were cultured on fresh TSA plates and incubated at 37 °C for 16–20 h. The MBC was defined as the concentration at which no viable bacterial growth was observed. The assay was performed in triplicate.

## In vitro biofilm studies

### Biofilm growth

*E. coli* ATCC 25922 and ATCC 35218 inoculum from freshly streaked agar plates were incubated for 18 h in LB broth supplemented with 0.2% glucose (or BHI for the Alamar blue assay) before being diluted in 0.90% saline to an OD_600_ of 0.50 ± 0.10. The suspensions were further diluted 1:100 in LB broth (or BHI broth for the Alamar blue assay) supplemented with 0.2% glucose and added to the wells of a 96-well plate. The last column was left with sterile LB broth (or BHI broth) as a negative control. The plates were wrapped in aluminium foil and incubated statically at 25 °C for 48 h to allow biofilm formation.

After incubation, the biofilms in the microtiter plate wells were washed twice with 0.9% saline to remove non-adherent bacterial cells. The biofilms were then exposed to 10 µg/mL GEN, either as an unformulated solution or loaded into MO-LCNPs, PHY-LCNPs, and liposomes at equivalent concentrations, and incubated for 24 h at 25 °C. After the 24 h treatment, the biofilms were washed twice with 0.9% saline.

### Crystal violet assay

To quantify biofilm biomass, biofilms were fixed onto the wells by adding 200 μL of methanol. After removing the methanol, the wells were stained with 0.1% (w/v) crystal violet for 15 min. Excess dye was washed away with deionised water, and the remaining dye that stained the biofilms was solubilised by adding 30% (w/v) acetic acid. The biofilm biomass was quantified by measuring the absorbance at 595 nm using a spectrometer (Inspire Multimode Plate Reader, PerkinElmer, Waltham, MA).

### Alamar blue assay

To quantify the viable bacterial count, a 10% Alamar blue solution was added to the biofilm wells, and the plate was immediately wrapped in aluminium foil to prevent evaporation and protect from light. The plate was then incubated at 25 °C on a 3D rotating platform at 70 rpm. Fluorescence was measured at 530 nm (excitation) and 590 nm (emission) every 30 min until the maximum intensity was reached.

### MBEC assay

The MBEC assay (Innovotech, Edmonton, Alberta, Canada) was used as a simplified in vitro model to compare different formulations of GEN. *E. coli* ATCC 25922 biofilm was grown as described previously. Briefly, an overnight culture of *E. coli* ATCC 25922 was adjusted to an OD_600_ of 0.50 ± 0.10 and diluted 1:100 in LB broth supplemented with 0.2% glucose. A 200 µL aliquot of the diluted bacterial suspension was transferred to the wells of the MBEC plates, and the peg lids of the plates were dipped into the suspension, with column 12 containing sterile LB broth as a negative control. The plates were incubated statically at 25 °C for 48 h to allow biofilm formation on the pegs.

### Minimum biofilm inhibitory concentration

The MBIC of GEN, GEN-LCNPs, and GEN-liposomes, was evaluated by the MBEC assay. *E. coli* ATCC 25922 biofilms were grown in MBEC plates as described earlier. Serial dilutions (200 µL) of unformulated GEN, GEN-LCNPs, and GEN-liposomes, with concentrations ranging from 0.78 to 400 μg/mL in LB broth, were added to a new 96-well plate. Following biofilm formation, the peg lid was rinsed in sterile PBS and inserted into the 96-well plate containing the serial dilutions of GEN formulations. The plate was incubated for an additional 18 h at 25 °C, after which the pegs were individually removed to quantify the remaining bacteria. The pegs were placed in 1 mL of sterile 0.9% saline solution and vortexed for 10 min to dislodge the bacteria. Serial dilutions were then prepared, and colony-forming units (CFU) were enumerated. The MBIC was defined as the first concentration that resulted in fewer than 99 bacterial colonies/mL. All experiments were conducted with three technical replicates across three separate occasions.

### Statistical analysis

All statistical analyses were performed using GraphPad Prism (version 8.1.2 for Windows; GraphPad Software, La Jolla, CA). Two-way ANOVA was used to assess the statistical significance of antimicrobial efficacy, followed by Tukey’s multiple comparison tests for the in vitro models. A P-value of < 0.05 or 0.01 was considered statistically significant. Data are presented as the mean ± standard deviation.

## Results & discussion

### Physiochemical characterisation

**Fig. 3:**
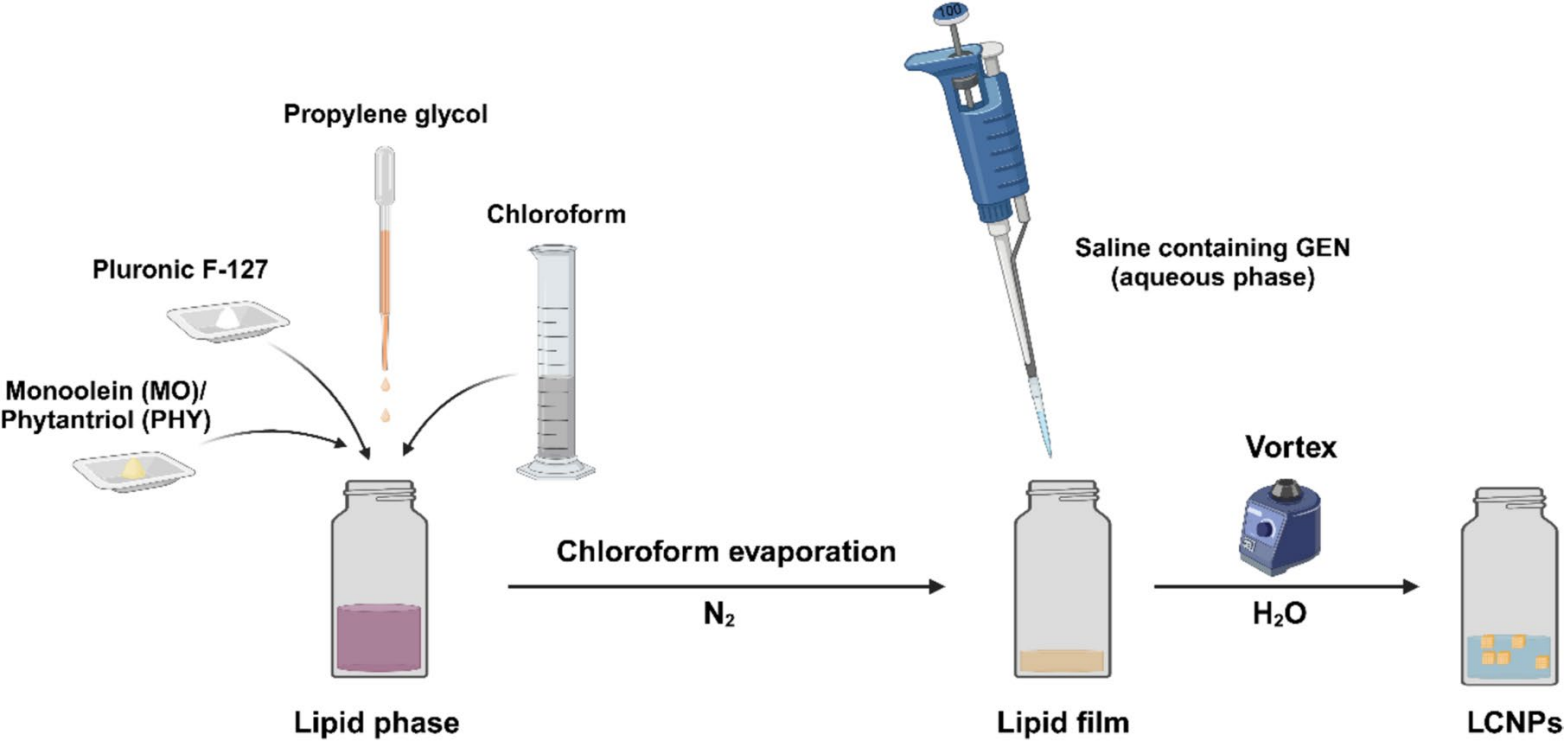
The fabrication scheme for GEN-LCNPs using the hydrotrope dilution method involves dissolving glycerol monooleate (MO), Pluronic^®^ F-127, and propylene glycol in chloroform. The chloroform is then evaporated under a stream of nitrogen gas. GEN in PBS is subsequently added to the lipid film, and the mixture is dispersed in Milli-Q water

MO- and PHY-LCNPs were prepared using the hydrotrope dilution method (Fig. 3), resulting in average particle sizes of 163 ± 3 nm and 178 ± 15 nm, respectively. In comparison, liposomes formed using Nanoassemblr with a DPPC: DPPG (1:1 w/w) ratio had a diameter of 152 ± 9 nm. The loading of GEN in both LCNPs (~ 170 nm) and liposomes (~ 185 nm) did not significantly affect the size of the lipid nanocarriers. The encapsulation efficiency of both LCNPs was approximately 95%, with a drug loading of 4.8% w/w, while the liposomes had an encapsulation efficiency of around 87% and a GEN loading of 3% w/w. The zeta potentials of unloaded MO-LCNPs, PHY-LCNPs, and liposomes were −25 mV, −24 mV, and −42 mV, respectively. After loading with GEN, the zeta potentials of MO-LCNPs, PHY-LCNPs, and liposomes decreased to −10 mV, −12.5 mV, and −38 mV, respectively, indicating the presence of some cationic GEN on the surface of the nanoparticles (Table 1).

**Table 1:** Particle size, PDI, and zeta potential of GEN unloaded, and GEN loaded MO-LCNPs, PHY-LCNPs and GEN-Liposomes. Data represent the mean ± standard deviation (*n* = 3)

	MO-LCNPs	GEN-MO-LCNPs	PHY-LCNPs	GEN-PHY-LCNPs	DPPC: DPPG Liposomes	GEN-Liposomes
Particle size (nm)	163 ± 30	172 ± 12	178 ± 15	172 ± 5	190 ± 15	185 ± 9
PDI	0.19 ± 0.02	0.2 ± 0.03	0.56 ± 0.02	0.51 ± 0.02	0.42 ± 0.02	0.36 ± 0.06
Zeta potential (mV)	−25.4 ± 0.5	−9.9 ± 0.2	−24 ± 0.7	−13.4 ± 0.2	−42 ± 0.5	−38 ± 0.3
Drug loading (mg/100 mg lipid, % w/w)	-	24.9 ± 0.2	-	24.7 ± 0.1	-	3.1 ± 0.2
Encapsulation efficiency (%)	-	95.4 ± 3	-	93.5 ± 2	-	82.7 ± 2

### Stability of lipid nanoparticles

**Fig. 4:**
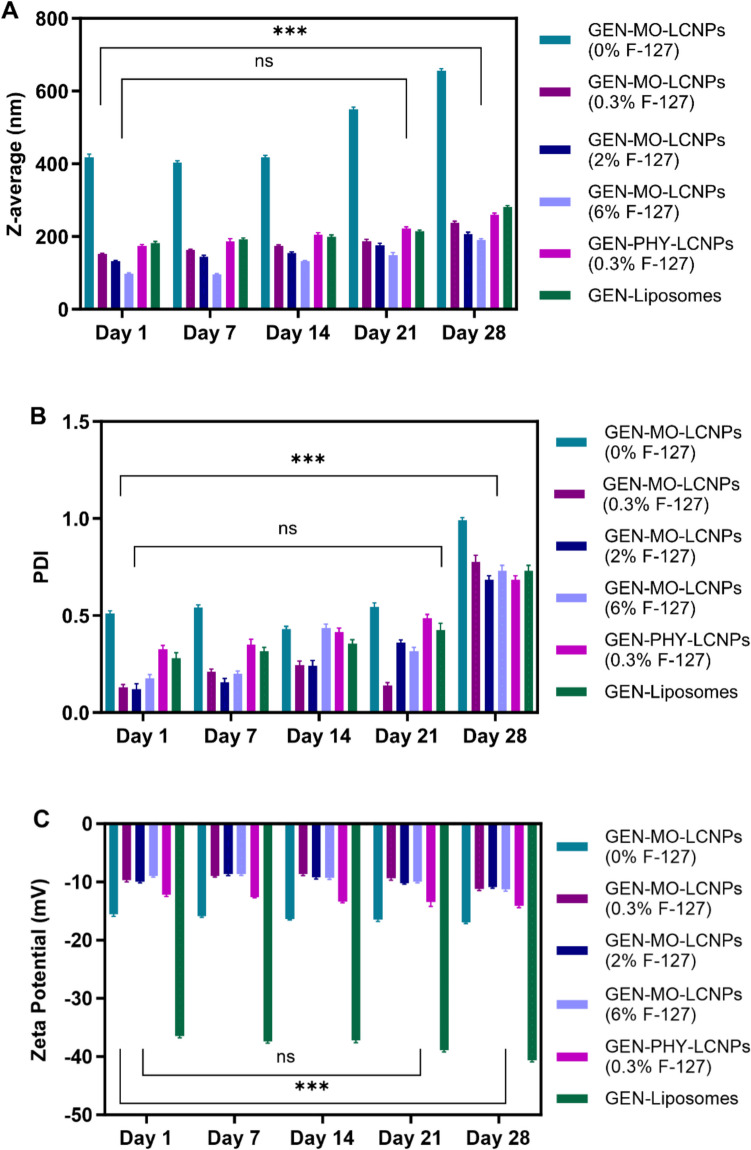
(**A**) Particle size, (**B**) PDI and (**C**) zeta potential of MO-LCNPs (with varying Pluronic^®^ F-127 concentrations), and PHY- LCNPs and liposomes over four weeks at 4 °C, n = 3

All lipid nanoparticles were monitored for stability over four weeks at 4 °C, assessing particle size, PDI, and zeta potential (see Fig. 4). GEN-loaded MO- and PHY-LCNPs exhibited well-defined particle sizes of 170 nm and 200 nm, respectively. PHY-LCNPs were significantly less stable than MO-LCNPs (except for MO-LCNPs without Pluronic^®^), often separating from suspension and requiring continuous dispersion through bath sonication before use. MO-LCNPs without Pluronic^®^ displayed a larger particle size compared to other MO-LCNPs with varying Pluronic^®^ concentrations, as the absence of Pluronic^®^ leads to more aggregation or larger clusters due to the lack of surfactant stabilisation (Fig. 4A). When Pluronic^®^ was added, it stabilised the nanoparticles, preventing aggregation and helping to maintain a smaller, more uniform size. [55]. The varying concentrations of Pluronic^®^ in MO-LCNPs allow for fine-tuning of particle size, with the optimal concentration resulting in the smallest and most stable nanoparticles [56].

Both LCNPs exhibited a negative zeta potential (< −10 mV), which alone does not provide sufficient electrostatic stability to maintain the dispersion. However, the presence of Pluronic^®^ provided steric hindrance, compensating for the weak electrostatic repulsion, stabilising the nanoparticles [57]. Liposomes of comparable size but with a reduced zeta potential were also formed (Fig. 4C). Typically, zeta potential values above ± 30 mV indicate strong electrostatic stability due to significant repulsive forces between particles [58]. Since the liposomes in this study had a zeta potential of > −36 mV, they are expected to remain stable in suspension without significant aggregation. No significant changes in the physicochemical parameters were observed over the first three weeks. However, by the fourth week, the particles showed slightly larger sizes, higher PDI values, and a small decrease in zeta potential (Fig. 4). Therefore, it can be concluded that all lipid nanoparticles remained stable for three weeks when stored at 4 °C.

### Nanoparticle tracking analysis and transmission electron microscopy

**Fig. 5:**
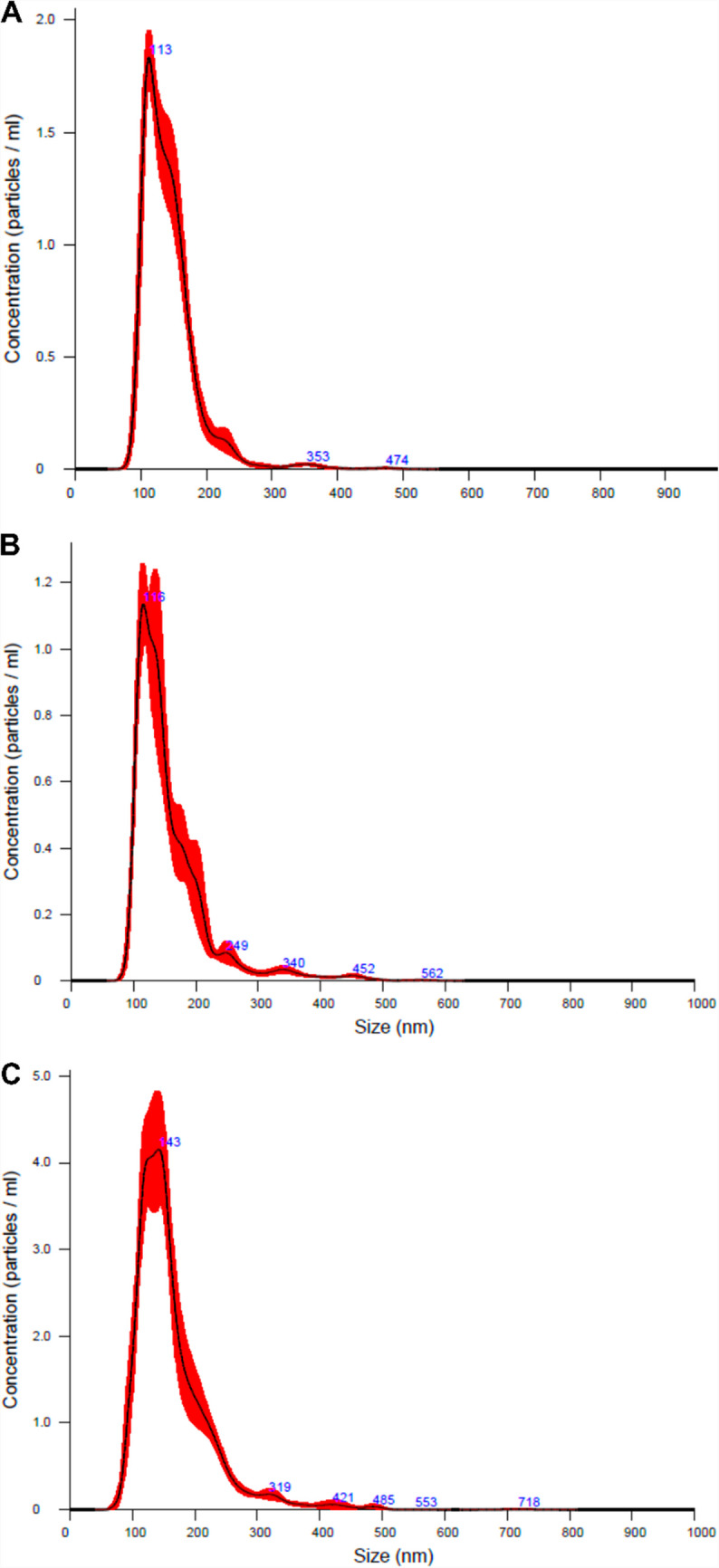
Nanoparticle tracking analysis (NTA) histogram demonstrating monodipersity and the average particle size of (**A**) GEN-MO-LCNPs (**B**) GEN-PHY-LCNPs (**C**) GEN-liposomes, n = 3

To further confirm the monodispersity, particle size, and concentration of LCNPs and liposomes, nanoparticle tracking analysis (NTA) was conducted. The average particle sizes of MO- and PHY-LCNPs were found to be 113 nm and 116 nm, respectively, with a narrow particle size distribution and an average particle concentration of 2 × 10^1^⁰ particles/mL (Fig. 5A, B). In comparison, the monodisperse liposomes exhibited an average particle size of 143 nm and a particle concentration of 8 × 10⁹ particles/mL (Fig. 5C).

**Fig. 6:**
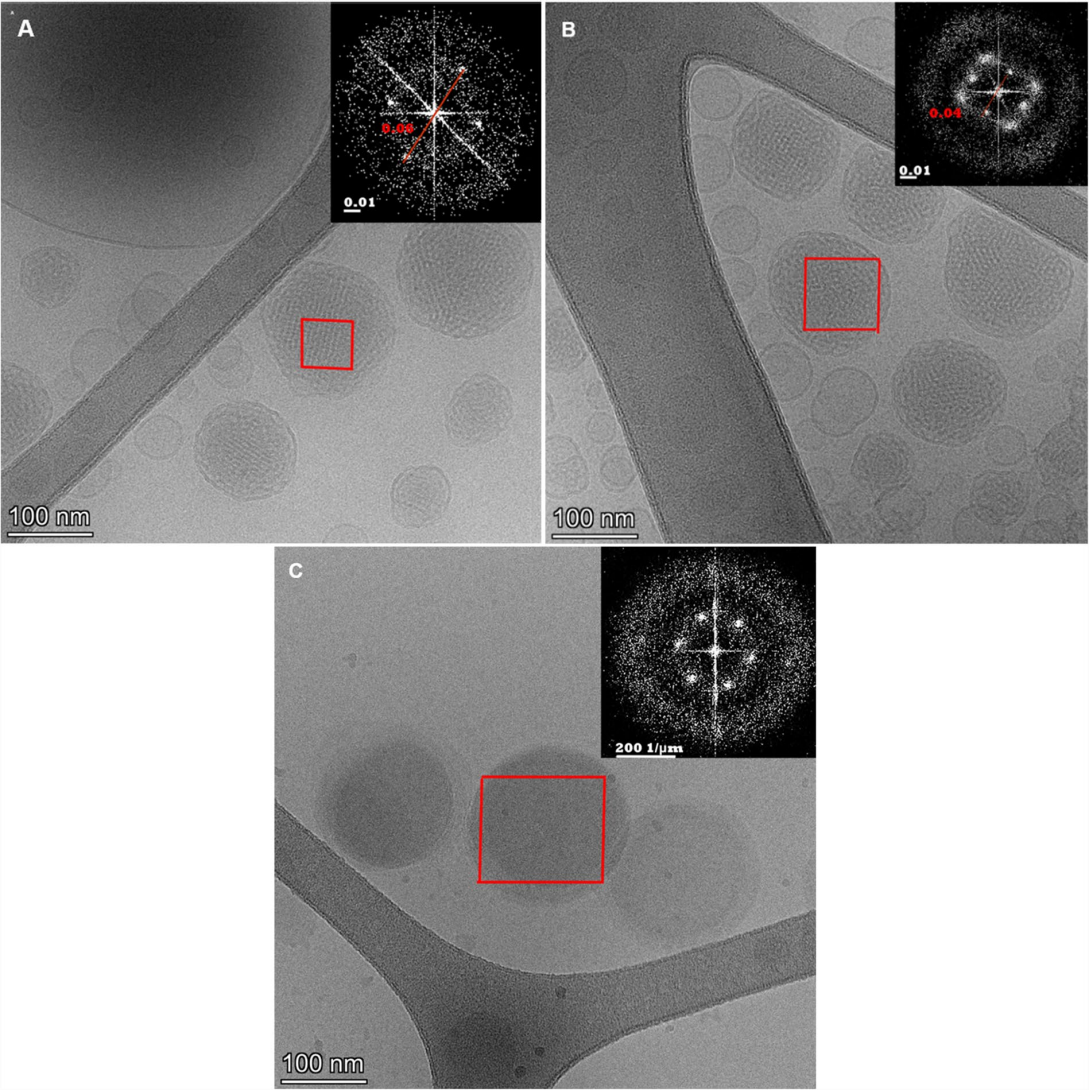
Cryo-TEM images (79,000 × 1125 resolution) and FFT analysis (inset) of (**A**) GEN-MO-LCNPs, (**B**) GEN-PHY-LCNPs (showing cubic-shaped lyotropic lipid liquid crystal nanoparticles with an ~ 8 nm interatomic lattice spacing), and (**C**) GEN-liposomes (demonstrating a unilamellar lipid bilayer structure with approximately 3 nm bilayer thickness)

Cryo-transmission electron microscopy (Cryo-TEM) images revealed both MO- and PHY-LCNPs to exhibita cubic morphology (cubosomes) (Fig. 6A, B), while the liposomes displayed a monolayer lipid bilayer structure with a diameter of approximately 160 nm and a bilayer thickness of around 3 nm, as confirmed by FFT analysis (Fig. 6C). The MO and PHY-LCNPs consisted of cubosomes with uniform size (~ 200 nm) and shape, displaying an ordered internal structure (cubic Pn3 m phase) that included a lattice interatomic spacing of approximately 8 nm, as determined by FFT analysis (Fig. 6A, B inset). This lattice spacing of around 8 nm is consistent with a lamellar or hexagonal phase structure, which is stable under certain conditions [59]. These findings align with the particle size data obtained from dynamic light scattering (DLS) and nanoparticle tracking analysis (NTA). The lattice interatomic spacing observed in the LCNPs likely reflects a nanostructured arrangement of lipid molecules within the nanoparticles'core [60]. This suggests that the nanoparticles possess a highly ordered or periodic structure, which could influence their stability, drug encapsulation, release properties, and overall performance in various applications. The observed spacing provides valuable insight into the molecular organisation of the LCNPs and may be linked to their structural integrity and functional properties [61].

Interestingly, these samples also contained a significant fraction of vesicles, which aligns with findings from previous studies [3, 4]. The presence of vesicles could provide additional functionalities, such as improved drug encapsulation or enhanced release properties. Previous research has shown that a large number of vesicles are present in GMO/F127-based dispersions prepared by microfluidisation or sonication [4–6]. It is believed that the presence of bilayer vesicles is essential for the cubosome formation process, which tends to occur slowly at room temperature [7]. To increase the proportion of cubosomes, a heat treatment cycle in an autoclave has been suggested as a potential approach [8]. However, this harsh treatment method, when applied to microfluidised samples, has previously resulted in phase separation or the formation of dispersions consisting only of large liposomes, as well as degradation of the encapsulated antimicrobial peptide.

### In vitro release studies

**Fig. 7:**
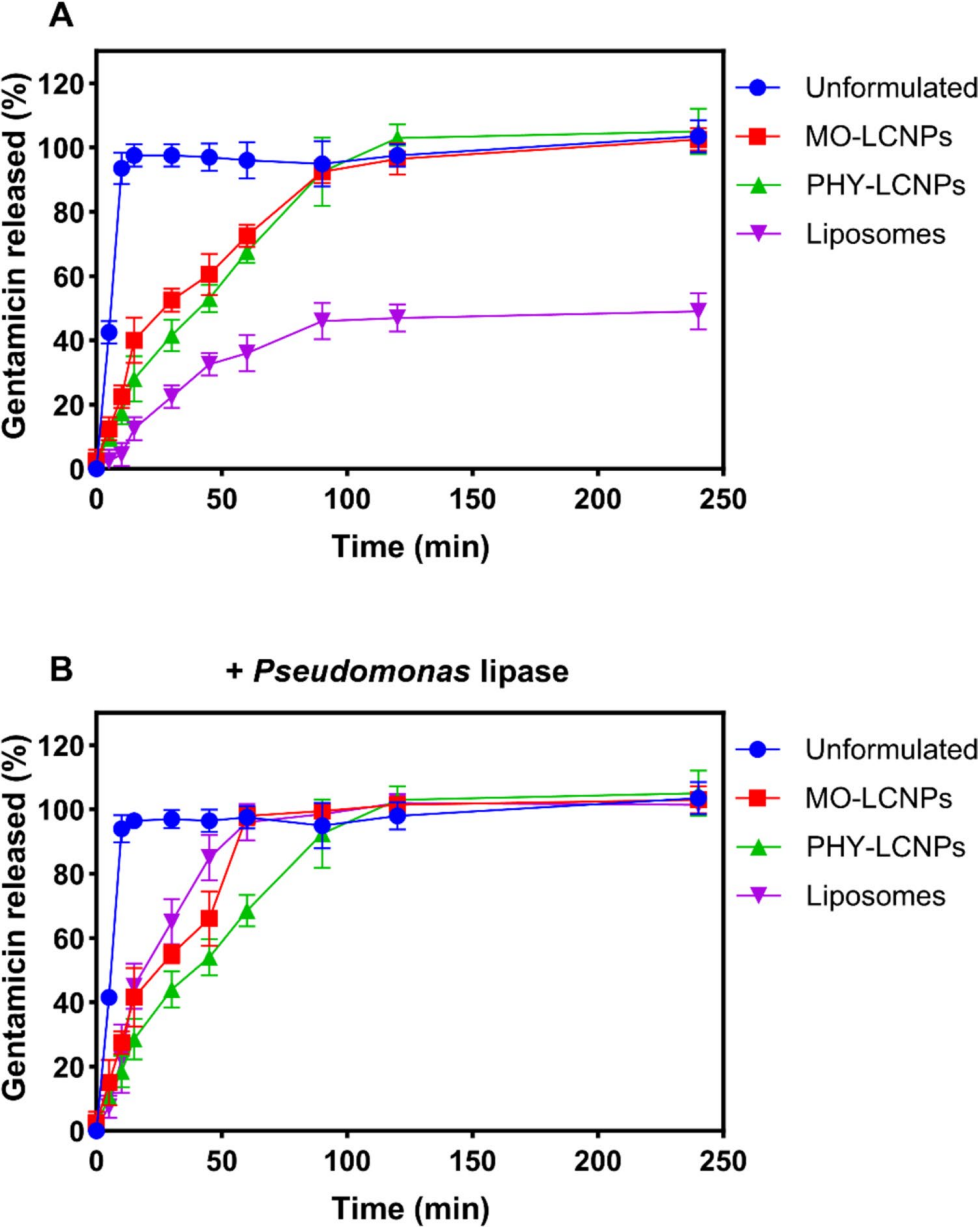
Gentamicin release from unformulated solution, MO-LCNPs, PHY-LCNPs, and liposomes in (**A**) PBS (pH 7.4) buffered media (**B**) with the addition of *Pseudomonas* lipase (1 mg/mL, 30 units/mL), n = 3

GEN was rapidly released from the LCNPs within 120 min, consistent with previous studies on TOB-loaded LCNPs [49]. Initially, the release rate of GEN from MO-LCNPs was higher than that from PHY-LCNPs. However, after reaching a plateau, both formulations exhibited comparable release profiles (Fig. 7A). The lipid liquid crystal matrix structure governs the release of the loaded antibiotics through diffusion [62]. The release rates of GEN from MO- and PHY-LCNPs were at least four times higher than the release from liposomes. The presence of bacterial lipase had minimal effect on the release of the small hydrophilic antibiotic GEN from LCNPs, with only a slight increase observed in digestible MO-LCNPs but not in the non-digestible PHY-LCNPs. This difference is attributed to the lipase-mediated digestion of the liquid crystalline structure of the monoglyceride (Fig. 7B). In liposomes, GEN release was limited to 43% in buffered solution, whereas bacterial lipase significantly accelerated the release to 92% within 60 min (Fig. 7B).

In a previous study, liposome formulations in PBS at 37 °C retained 75% of GEN over 48 h [63]. Maximum release of GEN occurred within 4 h of incubation at 37 °C, with the release remaining steady throughout the 48 h period. This is attributed to the osmotic equilibrium between the GEN concentration inside and outside the liposomes [64]. LCNPs, on the other hand, are 3D structures composed of multiple lipid bilayers, where water channels remain exposed to the external medium, unlike liposomes, which have a single lipid bilayer that fully encloses the internal aqueous compartment [48]. As a result, the rapid release of GEN from LCNPs was expected due to the unique configuration of these lipid nanoparticles.

### Antimicrobial efficacy against planktonic bacteria

**Fig. 8:**
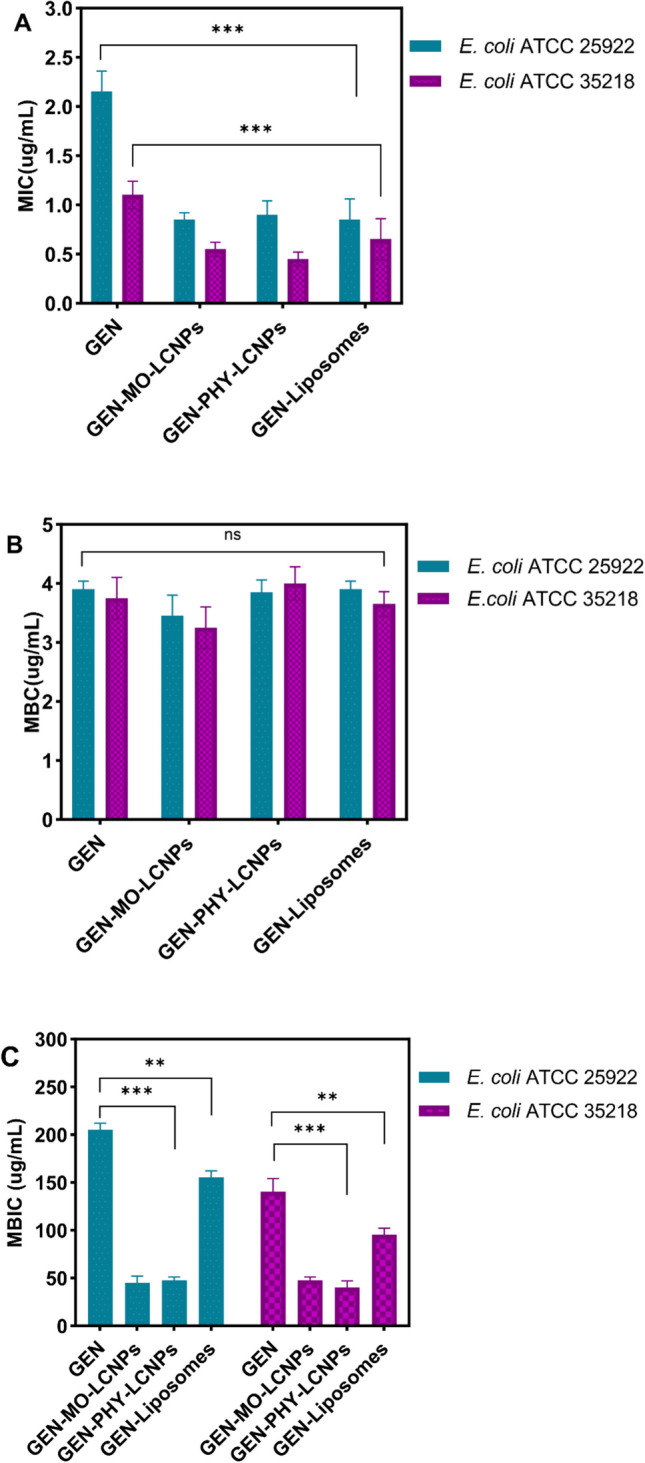
(**A)** Minimum inhibitory concentration (MIC) and (**B**) minimum bactericidal concentration (MBC) of unformulated GEN compared to GEN-MO-LCNPs, GEN-PHY-LCNPs, and GEN-liposomes against *E. coli* ATCC 25922 and *E. coli* ATCC 35218 strains in the planktonic state. (**C**) Minimum biofilm inhibitory concentration (MBIC) of unformulated GEN compared to GEN-MO-LCNPs, GEN-PHY-LCNPs, and GEN-liposomes against *E. coli ATCC* 25922 and *E. coli* ATCC 35218 strains in the biofilm state. Data are presented as mean ± standard deviation, n = 9, ***P < 0.0001, Student’s t-test

The antimicrobial activity of LCNPs and liposomes against two planktonic *E. coli* strains (ATCC 25922 and ATCC 35218) was assessed using the broth dilution method. In the planktonic state, 1 μg/mL and 2 μg/mL of unformulated GEN were required to inhibit the growth of *E. coli* ATCC 35218 and *E. coli* ATCC 25922, respectively (Fig. 8A). Interestingly, GEN-LCNPs demonstrated significantly lower MICs of 0.5 μg/mL and 1 μg/mL against *E. coli* ATCC 35218 and *E. coli* ATCC 25922, respectively, compared to unformulated GEN, showing a 1–2 dilution reduction in MIC values. GEN-liposomes exhibited antimicrobial activity comparable to that of GEN-LCNPs. However, no significant difference in the minimum bactericidal concentration (MBC) values was observed between unformulated GEN and GEN-loaded LCNPs and liposomes (Fig. 8B).

The interaction of LCNPs (and nanocarriers in general) with bacteria continues to be an active area of research [65]. The enhanced antimicrobial activity of GEN after encapsulation in lipid nanoparticles is primarily attributed to improved penetration and uptake of GEN by the bacteria, resulting in increased bacterial killing. Recently, Dyett et al. demonstratedthe uptake of individual cubosomes to occur, in part, through direct interaction between the cubosome lipids and the outer lipid plasma membrane [66]. This mode of interaction is further supported by findings from Boge [67], who observed cubosomes attaching to the bacterial interface of *E. coli*.

Additionally, Hong et al. recently demonstrated the direct interaction of cubic phase lipids with lipopolysaccharides [68], while Thorn et al. conducted fusion assays with *P. aeruginosa* [49]. The fusion efficiency of LCNPs with planktonic *P. aeruginosa* was 91.5%, indicating strong fusion potential, which could enhance antibiotic uptake by bacterial cells. In the present study, we hypothesise that the fusion capability is supported by the observed reduction in theMICof GEN against planktonic *E. coli.* These findings, along with other studies, generally highlight improved antibiotic loading performance [50, 69, 70]. Boge et al. reported that DPK-060 encapsulated in GMO-based LCNPs was particularly effective in inhibiting *E. coli* growth compared to the unformulated peptide, suggesting a synergistic effect where the encapsulation in cubic LCNPs contributes to enhanced antimicrobial activity beyond what the free peptide can achieve[71]. Furthermore, liposomal formulations of ciprofloxacin and GEN have been found to exhibit lower MICs and MBCs compared to their free drug counterparts against common resistant bacteria, including *E. coli*, *P. aeruginosa*, and *Klebsiella pneumoniae* [72]*.* The authors of these studies proposed that the enhanced antimicrobial efficacy of liposomal formulations is due to their effective and comprehensive contact with the bacterial cell’s outer membrane [73].

### Antimicrobial efficacy against biofilms

The antimicrobial efficacy of GEN was significantly different between planktonic and biofilm growth modes of *E. coli* (P < 0.0001). In the biofilm state, 200 μg/mL and 150 µg/mL of GEN were required to inhibit the internal bacterial community in *E. coli* ATCC 25922 and *E. coli* ATCC 35218, respectively (Fig. 8C). Both GEN-LCNPs resulted in a significant fourfold (for *E. coli* ATCC 25922) and threefold (for *E. coli* ATCC 35218) reduction in inhibitory concentrations in the biofilm state compared to unformulated GEN, with a MBICof 50 μg/mL (P < 0.0001), as shown in Fig. 8C. Liposomes demonstrated a twofold difference in MBIC values for both bacterial biofilms. A substantial body of literature suggests that lipid nanoparticles can enhance the efficacy of antimicrobial agents [23, 35, 74]. Due to their submicron size, surface properties, and biomimetic characteristics, lipid nanoparticles can penetrate through various biological barriers, such as biofilm matrices [39]. In this study, the enhanced effectiveness of GEN after encapsulation in LCNPs and liposomes mirrors the trend previously observed for tobramycin [31].

The limited antimicrobial effect of unformulated GEN observed in biofilms is due to its inability to target dormant, non-metabolically active bacteria [75]. The positively charged amino groups of GEN electrostatically bind to the negatively charged extracellular polymeric substances (EPS) of the biofilm, preventing it from reaching deeper bacterial layers, which hinders its ability to inhibit bacterial protein synthesis [76]. In contrast, GEN-loaded LCNPs overcame this electrostatic barrier at similar antibiotic concentrations. Previously, Thorn et al. demonstrated that LCNPs enhanced the distribution of tobramycin throughout the biofilm 3.5-fold, significantly improving antimicrobial activity [49]. The patch coating of LCNPs created a strong concentration gradient that promoted the direct penetration of antibiotics into the internal bacterial community, thereby increasing and enhancing their antimicrobial effect [49].

**Fig. 9:**
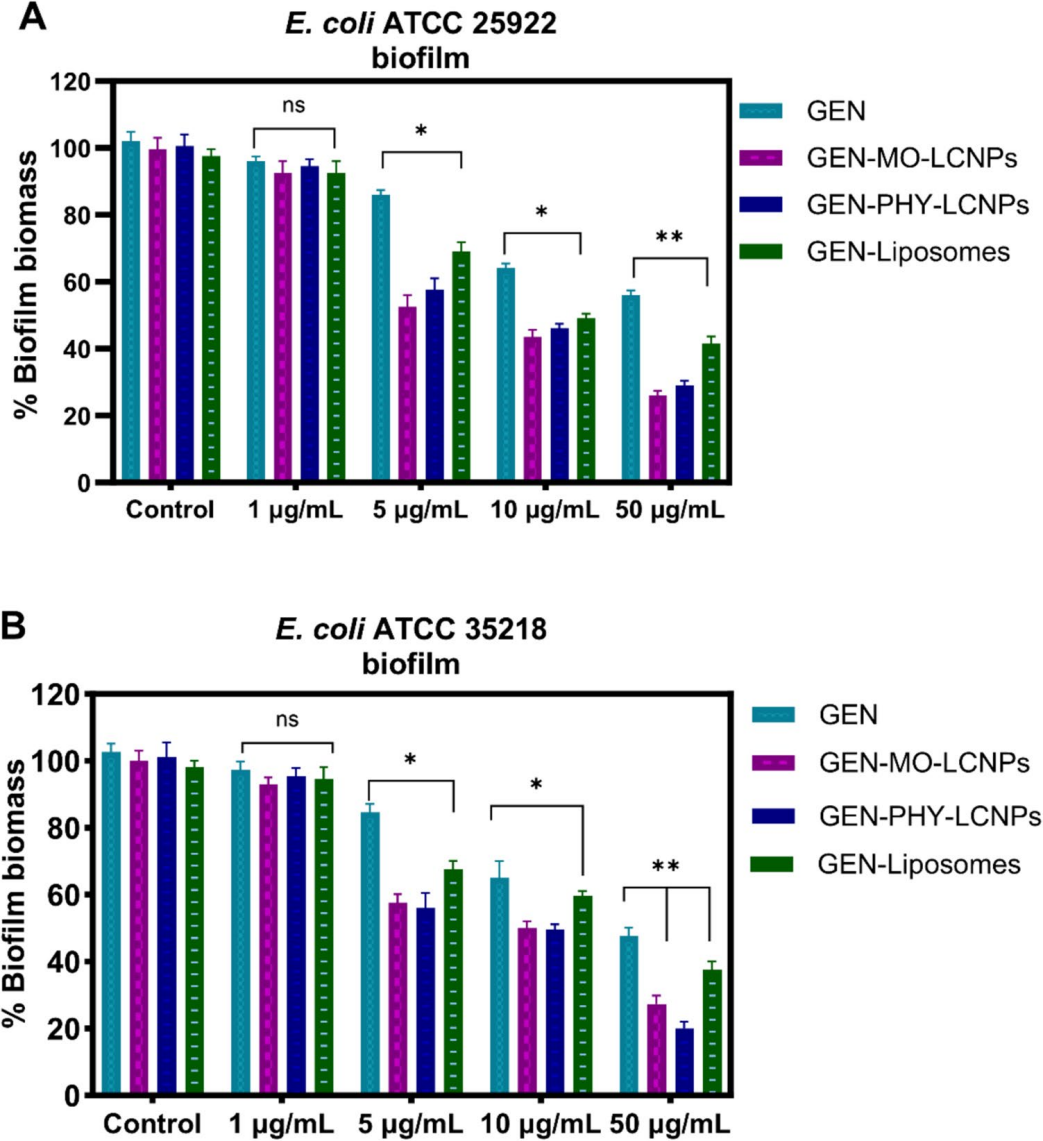
Biofilm biomass reduction of (**A**) *E. coli* ATCC 25922 and (**B**) *E. coli* ATCC 35218 strain biofilms grown on 96-well plates for 48 h and treated with varying concentrations of GEN, either as an unformulated solution or loaded into three different formulations: MO-LCNPs (0.04 mg/mL MO), PHY-LCNPs (0.04 mg/mL PHY), and liposomes (0.04 mg/mL DPPC: DPPG). Data are represented as mean ± standard deviation, n = 9 (3 independent experiments). Statistical analysis was performed using two-way ANOVA, followed by Tukey’s multiple comparison test. * = P < 0.01, ** = P < 0.001

GEN loaded into LCNPs, and liposomes demonstrated significantly enhanced antimicrobial effects against *E. coli* ATCC 25922 and *E. coli* 35,218 biofilms, as assessed by the crystal violet assay (Fig. 9). Compared to unformulated GEN, GEN-loaded MO-LCNPs, PHY-LCNPs, and liposomes reduced the biofilm biomass of *E. coli* ATCC 25922 by 19%, 23%, and 41%, respectively, over a range of GEN concentrations (Fig. 9A). All lipid formulations showed similar trends in antibiofilm activity against *E. coli* ATCC 35218 biofilms, with PHY-LCNPs performing better than MO-LCNPs, resulting in a 26% reduction in biofilm biomass.

**Fig. 10:**
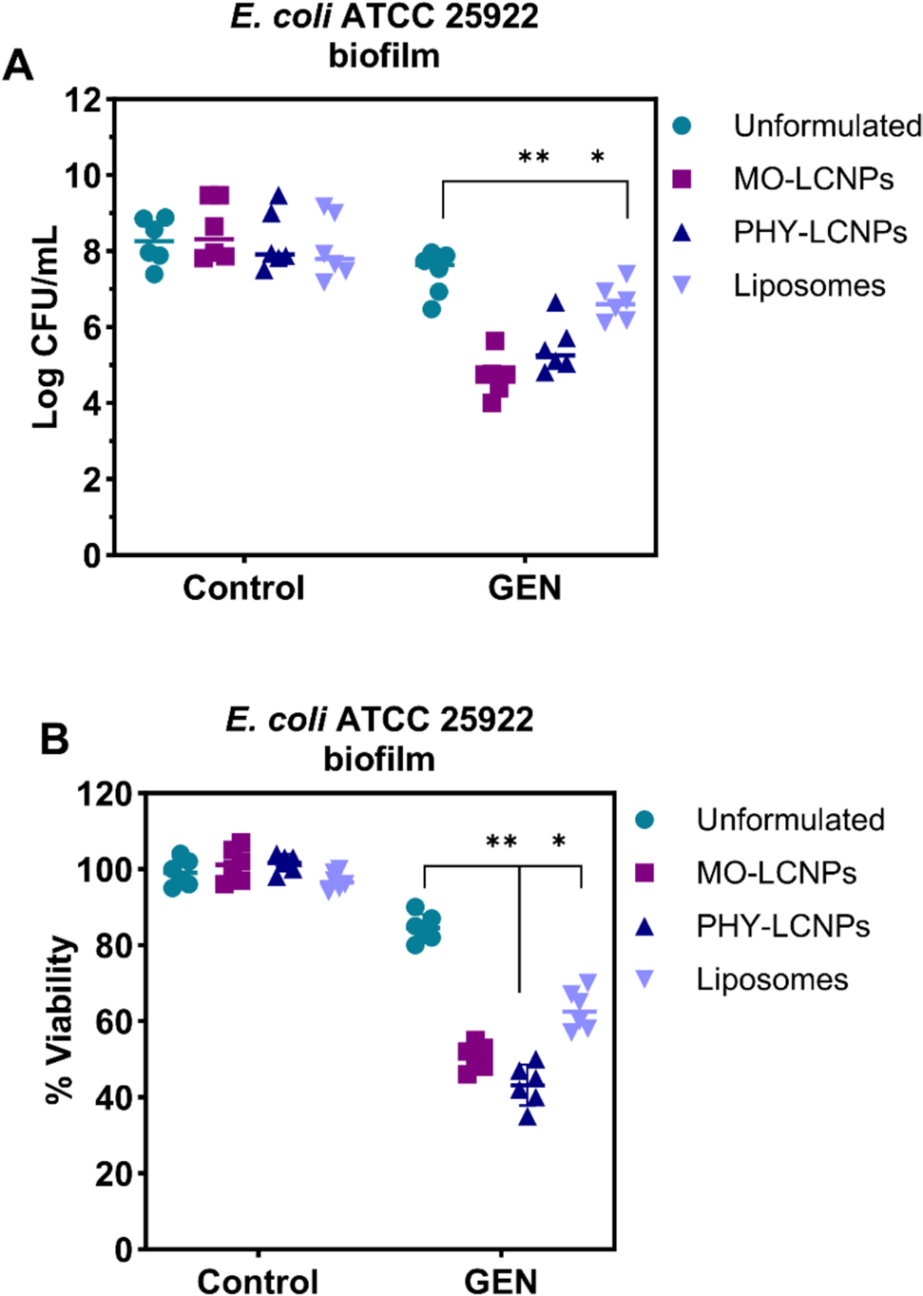
**A** Log10 CFU/mL of *E. coli* ATCC 25922 strain biofilms grown on MBEC plates for 48 h. **B** Percent viability of *E. coli* ATCC 25922 biofilms grown on 96-well plates and treated with GEN(10 μg/mL) either as an unformulated solution or loaded into three different formulations: MO-LCNPs (0.04 mg/mL MO), PHY-LCNPs (0.04 mg/mL PHY), and liposomes (0.04 mg/mL DPPC: DPPG). Data are presented as mean ± standard deviation, n = 6 (3 independent experiments). Statistical analysis was performed using two-way ANOVA, followed by Tukey’s multiple comparison test, * = P < 0.01, ** = P < 0.001

The antimicrobial effect of GEN against *E. coli* ATCC 25922 biofilms in the MBEC model was significantly enhanced when loaded into LCNPs. Compared to unformulated GEN and LCNPs without the drug, GEN-loaded LCNPs reduced the *E. coli* ATCC 25922 load by 5000-fold and 4000-fold, respectively, at the same GEN concentration (10 μg/mL) (Fig. 10A). However, liposomes with similar particle size did not significantly improve the antibacterial activity of GEN against MBEC-grown *E. coli* biofilms at identical concentration (10 μg/mL) (Fig. 9A). This suggests that the superior activity against *E. coli* biofilms is specific to LCNPs and not simply due to the size or lipid nature of the carrier. LCNPs likely facilitated better penetration of GEN through the biofilm matrix, improving bacterial targeting and enabling more sustained drug release, which supports prolonged antibiotic activity [23]. LCNPs exhibit superior antibacterial activity *E. coli* biofilms due to their unique internal nanostructure that enables sustained drug release and deeper biofilm penetration. Unlike liposomes, their deformable, non-lamellar structure allows better diffusion through the biofilm matrix and stronger interaction with bacterial membranes. This enhances local drug concentration and prolongs antibiotic action, making the effect specific to LCNPs [31]. These advantages over free GEN and liposomes indicate that LCNPs enhance the efficacy of antibiotics against biofilms, which are often resistant to conventional treatments [35].

The Alamar Blue assay, which measures cell viability, further confirmed that GEN-loaded LCNPs significantly reduce the viability of *E. coli* biofilms (Fig. 10B). This result supports the findings from the previous assays (CV, MBEC) and highlights the potential of LCNPs as effective carriers for enhancing the antimicrobial properties of drugs like GEN, especially against biofilm-associated infections [74].

**Fig. 11:**
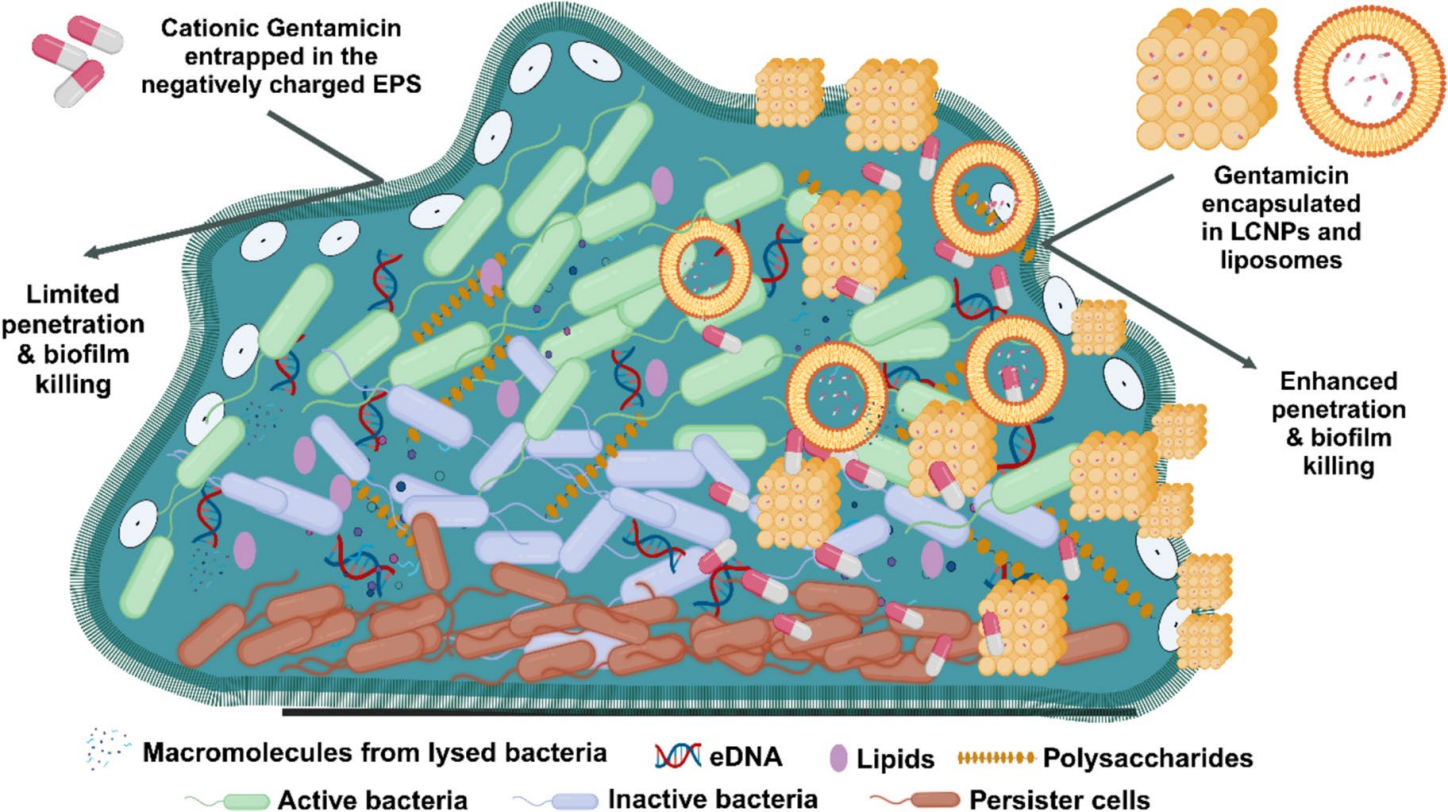
An illustration showing the enhanced efficacy of gentamicin through improved penetration and biofilm eradication following encapsulation in LCNPs and liposomes. The figure was created using Biorender.com

While liposomes have the potential to enhance the efficacy of antibiotics, particularly aminoglycosides, due to their fusogenic properties and surface characteristics [23] (Fig. 11), various phospholipid-based liposomes have been developed and patented as formulations to improve the activity of GEN, tobramycin, and amikacin [44, 63, 77, 78]. However, achieving maximum antibiotic efficacy against biofilms often requires specific phospholipid combinations. In our study, we found that compared to liposomes made from 1,2-dipalmitoyl-sn-glycero-3-phosphocholine (DPPC) and 1,2-dipalmitoylphosphatidylglycerol (DPPG), both MO- and PHY-LCNPs significantly enhanced the antimicrobial effect of GEN. The soft, 3D crystalline structure of the lipid bilayers in LCNPs, similar in composition, allows for fusion with biological lipid bilayers (e.g., cell membranes and biofilm matrices), which increases antibiotic uptake and enhances bacterial killing [65] (Fig. 11). Unlike unilamellar liposomes, the bilayers in LCNPs offer more efficient packing, providing a larger surface area for interaction with cells [79]. Additionally, LCNPs demonstrate improved skin retention compared to unilamellar liposomes, indicating that the complex lipid bilayer structure and increased localised curvature enhance permeability and facilitate better interaction with biological components. [80].

Interestingly, despite their different chemical properties, both MO- and PHY-LCNPs exhibited similar antimicrobial activity. At a GEN concentration of 10 µg/mL, the antimicrobial effect against *E. coli* ATCC 25922 biofilms were improved by approximately 5000-fold compared to unformulated GEN (Figs. 9 and 10). Although PHY is known for its antimicrobial properties [70], no intrinsic activity was observed at the concentrations used. The similarity in the liquid crystalline structures of MO-LCNPs and PHY-LCNPs may enhance their interaction with the biofilm matrix, promoting the formation of a"patch"that aids in driving the improved efficacy of GEN [41].

**Fig. 12:**
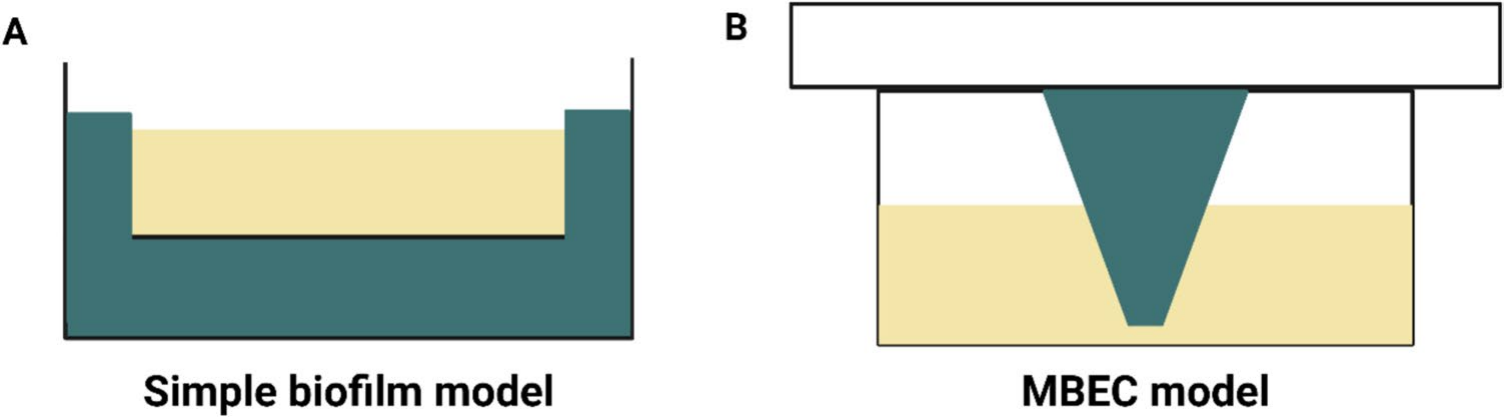
Representative illustrations of (**A**) Simple biofilm model and (**B**) MBEC model

The enhanced antimicrobial effect of GEN-LCNPs was consistent across the three in vitro biofilm models used in this study, including the MBEC model and two simple well-plate biofilm models (Figs. 9 and 10). In the simple biofilm model, bacterial cell viability was significantly reduced at 10 μg/mL GEN for GEN-MO-LCNPs (50%), GEN-PHY-LCNPs (41%), and GEN-liposomes (69%), compared to 80% bacterial viability after treatment with GEN solution (Fig. 10B). Unlike the simple biofilm model, where the biofilm forms at the bottom and side of the plate wells, the biofilm in the MBEC model is suspended on polystyrene pegs (Fig. 12), which reduces the potential bias caused by increased nanoparticle sedimentation [81]. Additionally, simple biofilms may differ structurally and metabolically from biofilms in the MBEC model. Regardless of the in vitro biofilm model used, loading GEN into LCNPs consistently enhanced its antibacterial effect.

Although LCNPs and liposomes have similar nanoparticle diameters, their surface zeta potentials differ, with LCNPs measuring − 10 mV and − 12.5 mV for MO and PHY, respectively, while liposomes have a more negative charge (− 36 mV). Messiaen et al*.* investigated that negatively charged nanoparticles tend to accumulate near biofilm cell clusters but are unable to effectively modulate antimicrobial activity due to repulsion from the negatively charged bacterial cell wall. [82]. In contrast, Thorn et al. found that the fusion efficiency of LCNPs with *P. aeruginosa* was enhanced, indicating that LCNPs do not repel bacterial cells. Instead, they adhere to the biofilm matrix in a patch-like formation, creating a steeper concentration gradient that promotes better penetration of tobramycin into the biofilm matrix [49]. Although the slightly negative surface charge of LCNPs might experience some repulsion from the biofilm matrix, their ability to permeate the biofilm matrix was sufficient to release GEN directly into the bacteria, increasing the overall concentration within the biofilm. Despite their slightly negative charge, LCNPs can still penetrate biofilms effectively due to their small size, non-lamellar structure, and surface flexibility. Their bicontinuous cubic phase allows them to deform and navigate through the dense, heterogeneous biofilm matrix. Additionally, stabilisers like Pluronic^®^ F127 can mask surface charge and reduce repulsion, while their high surface curvature minimizes nonspecific binding to EPS. These features collectively facilitate deep penetration and localized drug release near or inside bacterial cells [83]. Similarly, nanoparticles with slightly negative or near-neutral surface charges have been shown to improve targeting of biofilms compared to particles with highly negative or positive charges [39, 84]. In addition to particle size and surface charge, the superior antibiofilm efficacy of LCNPs compared to conventional liposomes may be attributed to their unique physicochemical characteristics. The solid crystalline lipid matrix of LCNPs provides a more rigid and stable nanostructure, which enhances drug encapsulation efficiency and promotes sustained release at the target site [85, 86]. This prolonged release profile allows for a continuous exposure of GEN to the bacterial cells embedded within the biofilm, thereby increasing the likelihood of disrupting the biofilm architecture and eradicating persistent bacterial populations. Moreover, the rigid nanostructure of LCNPs can facilitate deeper penetration into the dense EPS of biofilms, as they are less prone to structural deformation compared to more fluidic liposomal systems [87, 88]. The hydrophobic nature of the lipid core may also promote better interaction with the bacterial membrane, enhancing intracellular delivery [89]. These features collectively contribute to the enhanced therapeutic performance observed with LCNPs in this study.

### Effect of Pluronic^®^ coating on antimicrobial efficacy

**Fig. 13:**
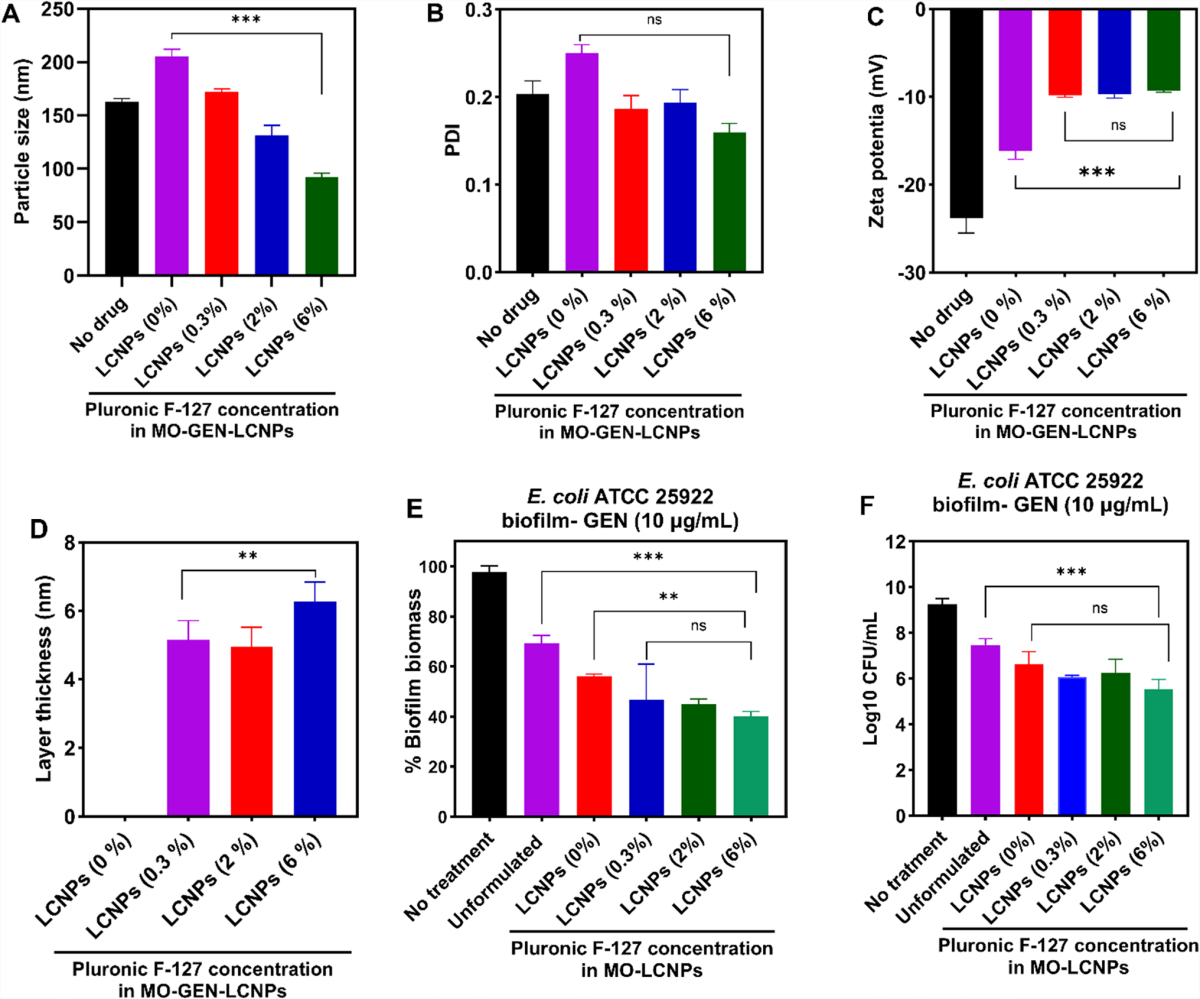
(**A**) Particle size, (**B**) PDI, and (**C**) zeta potential, and (**D**) adsorbed Pluronic^®^ F-127 layer thickness of blank MO-LCNPs and GEN-MO-LCNPs with varying Pluronic^®^ F-127 weight ratios. Antibiofilm activity of GEN in an unformulated solution and loaded into MO-LCNPs (0.04 mg/mL MO) with different concentrations of Pluronic^®^ F-127 (0–6%) against *E. coli* ATCC 25922 biofilms, as assessed by (**E**) crystal violet assay and (**F**) MBEC assay. Data are presented as mean ± standard deviation, n = 6 (3 independent experiments), two-way ANOVA, followed by Tukey’s multiple comparison test, ** = P < 0.01, *** = P < 0.0001

Surface functionalisation plays a critical role in determining the ability of particles to penetrate biological barriers [90]. Pluronic^®^ F-127, a triblock copolymer of PEG-PPO-PEG, acts as a steric stabiliser for various mesophase particles, including MO-cubosome nanoparticles, preventing aggregation [91]. It has also been shown to significantly enhance the permeability of sub-300 nm organic, polymeric, and lipid nanoparticles across different biological barriers [92–94]. Since the biofilm extracellular polymeric substance (EPS) matrix acts as both a physical and biological barrier to nanoparticle penetration, we formulated GEN-loaded MO-LCNPs with varying Pluronic^®^ F-127 weight ratios to investigate its effect on antimicrobial activity while maintaining a constant GEN loading. The original MO-LCNPs made with 0.3% (w/v) Pluronic^®^ F-127 had a particle size of ~ 174 nm. Increasing the polymer content significantly reduced the particle size of the LCNPs to approximately 95 nm, while LCNPs without Pluronic^®^ F-127 exhibited significantly larger particle sizes (Fig. 13A). All MO-LCNPs exhibited similar zeta potentials (−10 mV), except for the MO-LCNPs without Pluronic^®^, which had a significantly higher zeta potential (−16 mV), with polydispersity indices (PDIs) around 0.2 for all formulations (Fig. 13B, C).

We hypothesised that tuning the density of the Pluronic^®^ F-127 coating could impact the biofilm permeability of the LCNPs, thereby affecting their antimicrobial efficacy. However, this effect was not observed. To explore this, we assessed the thickness of the Pluronic^®^ F-127 coating adsorbed onto the MO-LCNPs surface at varying Pluronic concentrations using zeta potential measurements. The adsorbed Pluronic^®^ F-127 forms a uniform layer around the nanoparticles, which shifts the slipping plane and alters the zeta potential. The difference in zeta potential before and after coating provides an indirect measure of the polymer layer thickness [95].

MO-LCNPs without any Pluronic^®^ (zero adsorbed layer thickness) exhibited the highest zeta potential value (Fig. 13D) indicating a higher surface charge compared to the Pluronic^®^ -coated formulations. When comparing MO-LCNPs prepared using 0.3%, 2%, and 6% w/v Pluronic^®^ F-127, no significant differences in zeta potential values were observed, suggesting that the coating thickness across these concentrations was similar (Fig. 13D). This implies that the Pluronic^®^ F-127 coating, within the concentrations used in this study, did not substantially affect the overall surface charge or particle stability of the MO-LCNPs. Interestingly, despite no significant difference in the zeta potential and particle size across Pluronic^®^ concentrations, GEN-MO-LCNPs demonstrated a significantly greater reduction in biofilm biomass (Fig. 13E) and bacterial load (Fig. 13F) compared to the unformulated GEN solution. This indicates that the enhanced antimicrobial activity of GEN is mainly attributed to the liquid crystal structure of the LCNPs, rather than variations in the density of the Pluronic^®^ F-127 coating. The liquid crystalline structure of LCNPs is known for its unique ability to interact with biological membranes, including bacterial biofilms, allowing for increased penetration and sustained release of the drug, which in turn enhances bacterial killing. This is supported by previous studies that have shown LCNPs to be highly effective in targeting and disrupting bacterial biofilms, which are often highly resistant to conventional treatments [39].

However, Pluronic^®^ F-127 concentrations in the range of 5–15% w/w are commonly utilized in LCNPs for drug delivery applications, as they offer a favourable balance between nanoparticle stability and controlled drug release. However, it has been reported that Pluronic^®^ concentrations exceeding 15% in PLGA nanoparticle coatings can lead to instability, fluctuations, and disruption of the nanoparticle structure, which would impair their performance in drug delivery systems [95]. Therefore, maintaining the Pluronic^®^ F-127 concentration within an optimal range is crucial to ensuring the stability and functional integrity of the nanoparticles.

Overall, these findings suggest that the antimicrobial activity of GEN is enhanced through encapsulation in MO-LCNPs, and the primary factor contributing to this enhanced activity is the inherent properties of the LCNPs, such as their liquid crystal structure and ability to interact with bacterial biofilms. The role of the Pluronic^®^ F-127 coating, within the concentrations tested, appears to be minimal in influencing the antibacterial effect, further highlighting the importance of the LCNPs'unique structure in improving the efficacy of antibiotic treatments against biofilm-associated infections.

## Conclusion

In the current research, we investigated the potential of liquid crystal nanoparticles (LCNPs) and liposomes as advanced drug delivery systems for GEN to combat *E. coli* biofilms. The findings demonstrate that LCNPs significantly enhance the antimicrobial efficacy of GEN against *E. coli* biofilms compared to traditional liposomal formulations. TEM confirmed the stability and distinct cubic structure of LCNPs, with a particle size around 200 nm, while liposomes exhibited a monolayer lipid bilayer structure with a diameter of approximately 160 nm. All lipid nanoparticles (MO-, PHY-LCNPs, and liposomes) retained stability over three weeks of cold storage, suggesting their potential for practical use.

The antimicrobial study findings highlight that LCNPs provide a two-fold reduction in the MIC values and a substantial four-fold reduction in inhibitory concentrations in biofilm states compared to unformulated GEN. In contrast, liposomes only achieved a two-fold reduction inMBIC values. LCNPs also resulted in a significant reduction in bacterial colony-forming units (CFU) by 5000-fold for *E. coli* ATCC 25922, underscoring their superior efficacy in penetrating biofilm matrices and enhancing drug delivery.

Moreover, the study demonstrates that the enhanced performance of LCNPs is independent of particle size and Pluronic^®^ concentration, indicating that the structural composition of LCNPs plays a critical role in their effectiveness. These results suggest that LCNPs could serve as a promising platform carrier for antibiotics like GEN, particularly in treating biofilm-related infections where conventional antibiotics often fail due to limited penetration into the biofilm matrix. This advancement in nanocarrier technology holds significant potential for improving clinical outcomes in treating resistant bacterial infections.

## Data Availability

The datasets generated during and/or analysed during the current study are available from the corresponding author on reasonable request.
